# Supplementary material for: The neural encoding of information prediction errors during non-instrumental information seeking
Source: Sci Rep. 2018 Apr 17;8:6134. doi: 10.1038/s41598-018-24566-x (PMC5904167; doi:10.1038/s41598-018-24566-x)
Supplement: Supplementary file 1 — Supplementary Information [file 41598_2018_24566_MOESM1_ESM.pdf]

SUPPLEMENTARY MATERIALS

The neural encoding of information prediction errors during non-instrumental information seeking

Maja Brydevall[1,2,+], Daniel Bennett[1,2,+], Carsten Murawski[2] & Stefan Bode[1]

[1] School of Psychological Sciences, The University of Melbourne, Parkville, Victoria 3010, Australia

[2] Department of Finance, The University of Melbourne, Parkville, Victoria 3010, Australia

[+] these authors contributed equally to this work

Supplementary Table 1. Number of participants included in each analysis.

Behavioural analyses	RPE (informative stimulus)	IPE (informative stimulus)	Amount of information (informative stimulus)	RPE (non-informative stimulus)	IPE (non-informative stimulus)	Amount of information (non-informative stimulus)	Outcome screen (all)	Outcome screen (informative stimulus)	Outcome screen (non-informative stimulus)
22	15<br>[162.1 ± 93.6]	15<br>[86.1 ± 49.9]	15<br>[162.1 ± 93.6]	19<br>[257.8 ± 64.2]	19<br>[143.5 ± 35.2]	19<br>[257.8 ± 64.2]	19<br>[79.4 ± 20.0]	19<br>[30.8 ± 20.0]	18<br>[48.7 ± 26.0]

RPE: reward prediction error. IPE: information prediction error. For ERP analyses, values in parentheses denote mean number of epochs included in analysis ± 1 standard deviation.

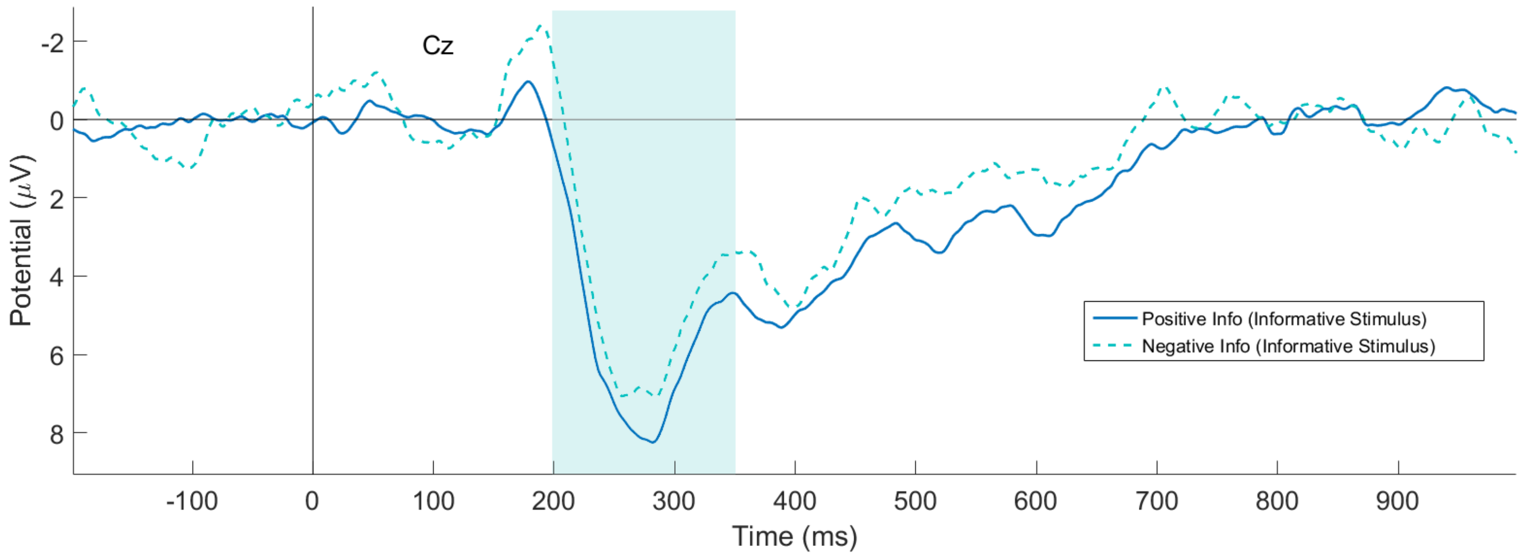

Supplementary Figure S1. Grand average ERP waveforms at electrode Cz for positive and negative amount of information in the informative stimulus. The teal rectangle denotes the FRN measurement window (200–350ms). Time 0 denotes the presentation of a card stimulus.

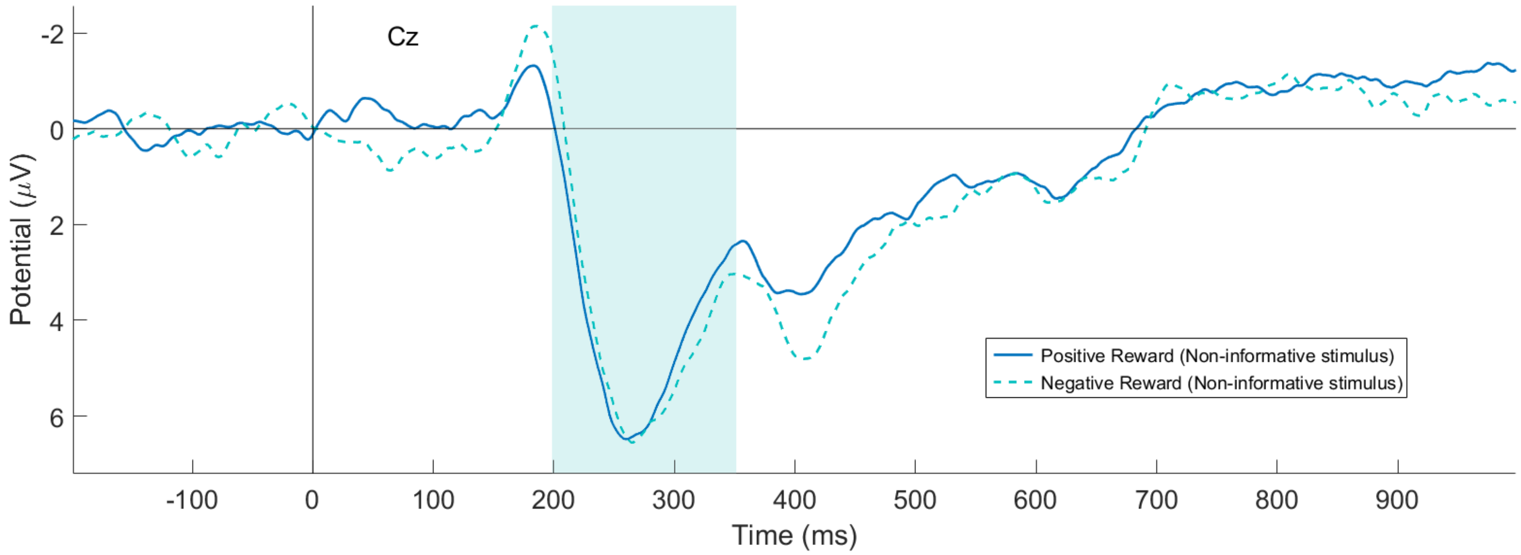

Supplementary Figure S2. Grand average ERP waveforms at electrode Cz for positive and negative reward prediction errors in the non-informative stimulus. The teal rectangle denotes the FRN measurement window (200–350ms). Time 0 denotes the presentation of a card stimulus.

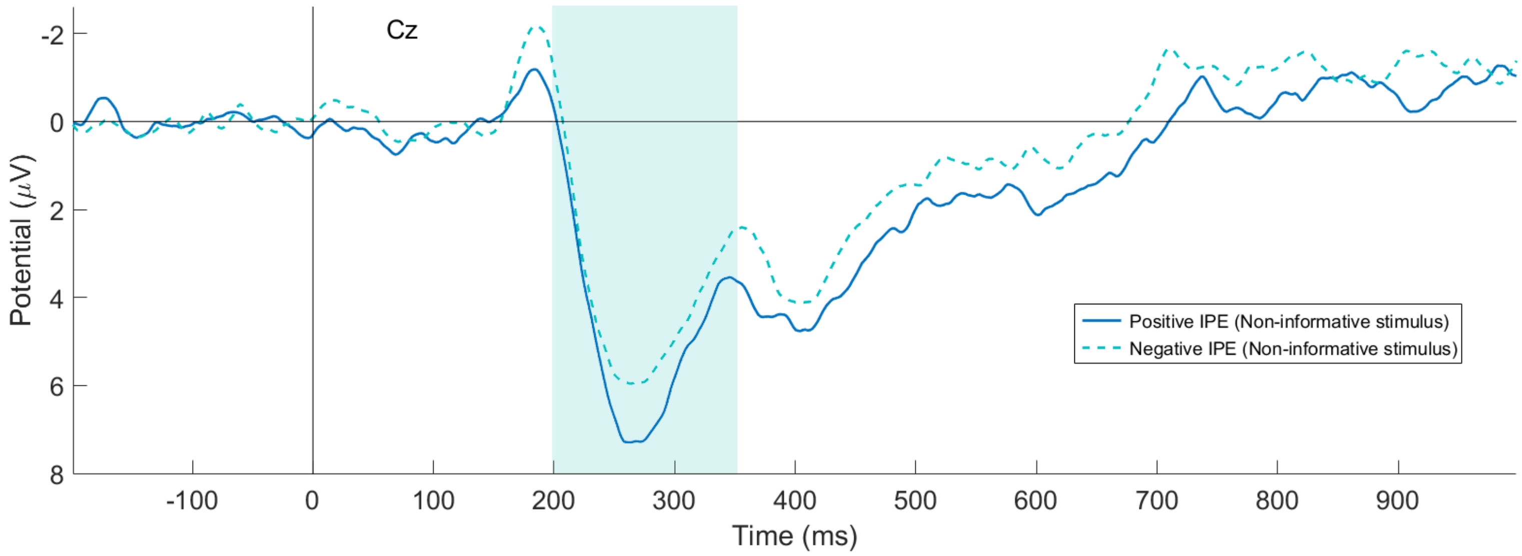

Supplementary Figure S3. Grand average ERP waveforms at electrode Cz for positive and negative information prediction errors in the non-informative stimulus. The teal rectangle denotes the FRN measurement window (200–350ms). Time 0 denotes the presentation of a card stimulus.

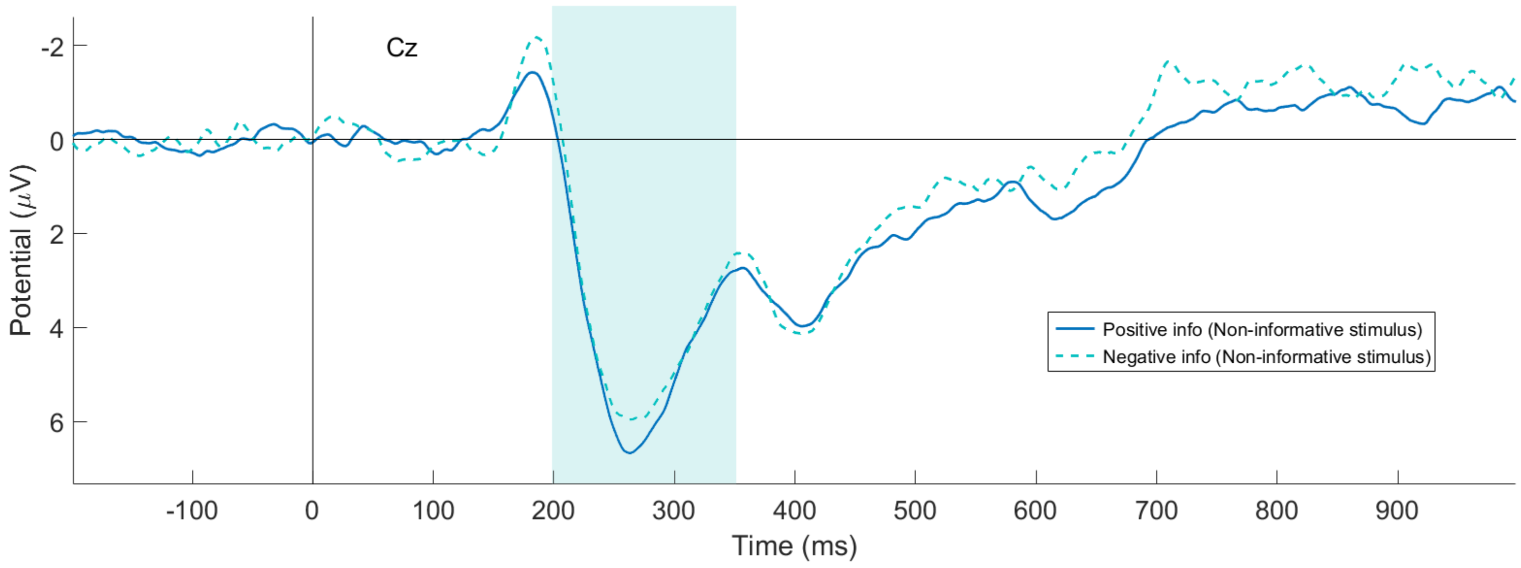

Supplementary Figure S4. Grand average ERP waveforms at electrode Cz for positive and negative amount of information in the non-informative stimulus. The teal rectangle denotes the FRN measurement window (200–350ms). Time 0 denotes the presentation of a card stimulus.

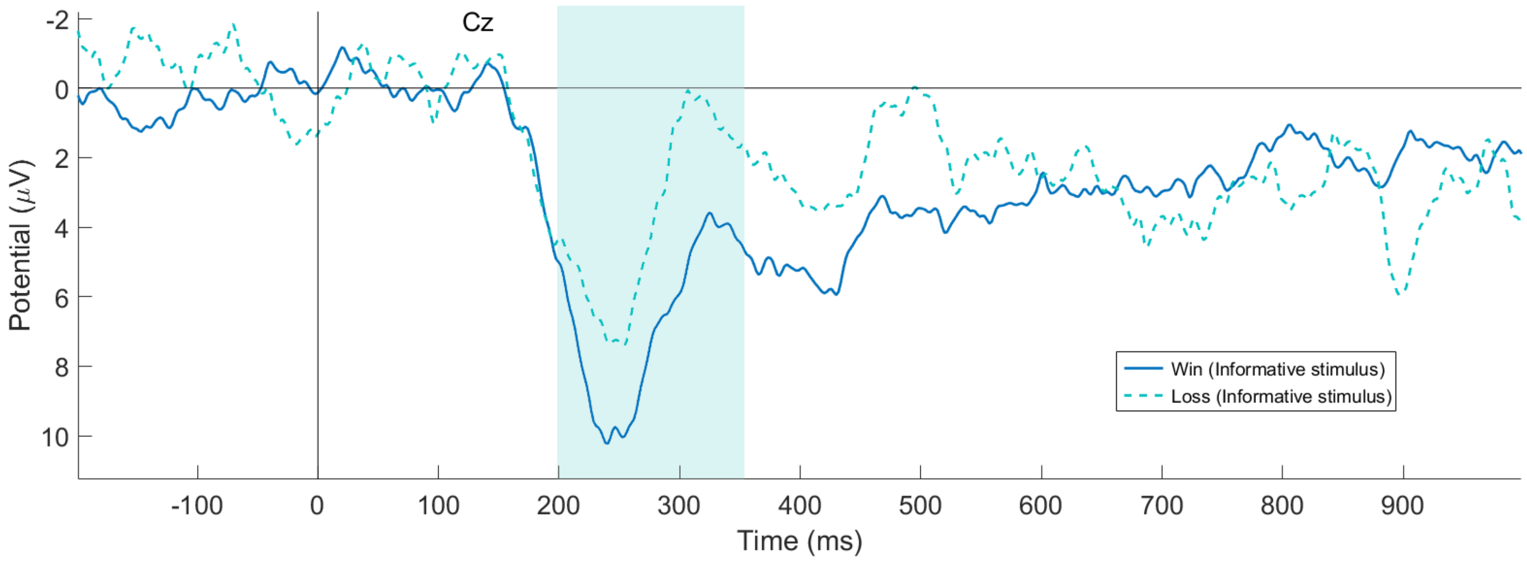

Supplementary Figure S5. Grand average ERP waveforms at electrode Cz for win and loss outcomes following an informative stimulus. The teal rectangle denotes the FRN measurement window (200–350ms). Time 0 denotes the presentation of an outcome screen.

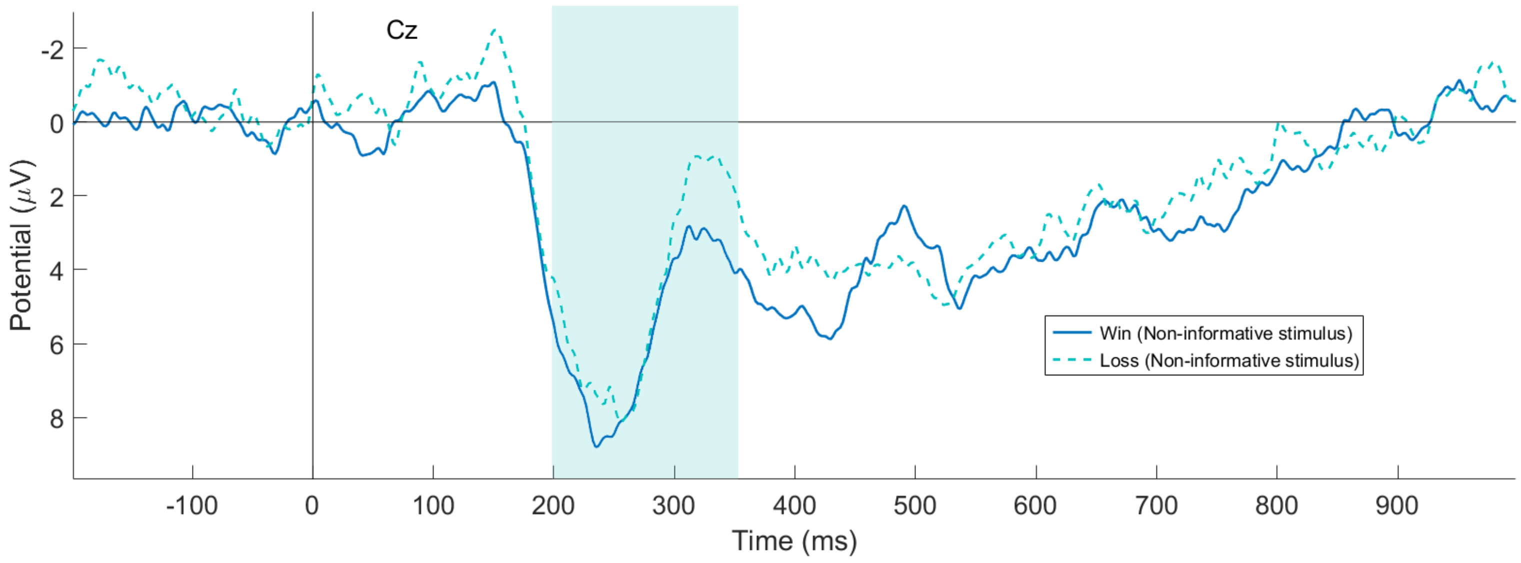

Supplementary Figure S6. Grand average ERP waveforms at electrode Cz for win and loss outcomes following a non-informative stimulus. The teal rectangle denotes the FRN measurement window (200–350ms). Time 0 denotes the presentation of an outcome screen.
